# BEHAVIORAL HEALTH INTERVENTIONS IN THE HOSPICE SETTING: A SCOPING REVIEW

**DOI:** 10.1093/geroni/igad104.3011

**Published:** 2023-12-21

**Authors:** Lilla Brody, Karolina Sadowska, Alexis Holllingsworth, Michael Ong, Tejas Subramanian, Drew Wright, M Cary Reid, Daniel Shalev

**Affiliations:** Weill Cornell Medicine, Washington, District of Columbia, United States; Weill Cornell Medicine, New York City, New York, United States; Weill Cornell Medical College, New York City, New York, United States; Weill Cornell Medical College, New York City, New York, United States; Weill Cornell, New York City, New York, United States; Weill Cornell Medical College, New York City, New York, United States; Weill Cornell Medicine, New York City, New York, United States; Weill Cornell Medicine, New York City, New York, United States

## Abstract

Hospice is a model of care for individuals with a life expectancy of less than six months who choose to focus their treatment on quality-of-life rather than extending life. In 2019, half of all Medicare decedents elected to receive hospice care. Behavioral health comorbidities are prevalent in hospice patients; approximately 40% experience significant depression and/or anxiety. Such comorbidities are associated with poor outcomes including increased physical symptoms, worse function, and lower quality-of-life. Despite the impact of behavioral health comorbidities on hospice patient outcomes, little research has focused on the use of behavioral health interventions in the hospice setting. We conducted a scoping review to characterize the types of behavioral health interventions administered to patients receiving hospice care. We searched four databases, generating 6,842 unique citations over any time period. 70 texts were reviewed and 31 were retained for analysis. Studies examined both psychotherapeutic (n=10) and psychopharmacologic (n=10) interventions, as well as other non-pharmacologic interventions (n=11). Duration of study follow-up was available for 17 of the studies, ranging from six days to six months. 14 studies reported information on interventionists, who were most commonly music (n=3) or massage therapists/acupuncturists (n=3). Average interventions were 47 minutes for six sessions. 18 studies presented statistically significant results related to primary outcomes, most commonly depression (n=9), anxiety (n=6), and pain (n=4). Our findings demonstrate a dearth of behavioral health interventions targeting hospice patients, suggesting the need for increased research and clinical focus on developing, evaluating, and implementing behavioral health interventions in the hospice setting.

